# ADDRESSING UNMET LONG-TERM SERVICES AND SUPPORTS NEEDS FOR RACIAL/ETHNIC MINORITY OLDER ADULTS

**DOI:** 10.1093/geroni/igac059.742

**Published:** 2022-12-20

**Authors:** Jasmine Travers

**Affiliations:** NYU, New York City, New York, United States

## Abstract

The COVID-19 pandemic magnified several long-standing problems with the delivery of long-term services and supports, including access to care in the community setting. A disproportionate rise in nursing home use among Black and Latino older adults reflects the inadequacy of existing programs and policies to support aging in place for these most at-risk populations. Enabling aging in the community and preventing avoidable nursing home placements is widely considered a priority by federal, state, and local entities along with families and older adults. Yet, it is unclear what is needed to support Black and Latino older adults to remain in the community. In this presentation, Dr. Travers will discuss unmet long-term services and supports needs among the Black and Latino population, issues particularly faced by these populations during COVID-19, and opportunities to move forward as we transition to an 'endemic' COVID-19 landscape.

